# FORETHOUGHT OR AFTERTHOUGHT? THE FUTURE OF GERONTOLOGICAL EDUCATION

**DOI:** 10.1093/geroni/igad104.2114

**Published:** 2023-12-21

**Authors:** Julie Masters, Mark Staley, Rona Karasik

**Affiliations:** University of Nebraska Omaha, Omaha, Nebraska, United States; University of Nebraska - Omaha, Omaha, Nebraska, United States; Saint Cloud State University, Saint Cloud, Minnesota, United States

## Abstract

The increasing population of older adults would suggest a comparable increase in the demand for gerontological education. The current workforce shortage and number of Gerontology programs that are struggling and/or closing, however, suggest this is not necessarily the case. Bass and Ferraro (2000) reported over 1,000 gerontology/aging or related programs in operation. Today, there are 537 programs in gerontology and/or aging including 31 Associate degrees; 185 Minors; 79 Undergraduate and 92 Graduate Certificates; 43 Majors; 57 Masters; and 16 doctoral programs (University of Nebraska Omaha, 2023). Many interrelated factors have been suggested to account for the decline. From an economic point of view, these factors are thought to include: decreasing enrollments in and revenue generation from gerontology programs; demographic shifts away from traditional age college students; and decreasing interest and value of higher education overall. From a pedagogical point of view, this might reflect a return to individual courses and programs ensconced in more traditional disciplinary approaches (e.g., family studies, psychology, and sociology). Pervasive ageism has also been thought to be an underlying societal factor. While none of these factors are easily remedied, understanding their impact is an important first step. GSA programming such as Ageism First Aid and Reframing Aging Initiative are taking the next Herculean step in tackling societal ageism. This presentation focuses on reframing the value of gerontological education to students, employers, and policymakers– moving it from an afterthought to a prioritized strategy for addressing the mounting demand for aging services and service providers.

